# Oral Rehabilitation for Amniotic Band Syndrome: An Unusual Presentation

**DOI:** 10.5005/jp-journals-10005-1283

**Published:** 2015-04-28

**Authors:** Kavita Hotwani, Krishna Sharma

**Affiliations:** Senior Lecturer, Department of Pediatric and Preventive Dentistry, VSPM’s Dental College and Research Centre, Nagpur, Maharashtra, India; Senior Lecturer, Department of Orthodontics and Dentofacial Orthopedics Sharad Pawar Dental College, Wardha, Maharashtra, India

**Keywords:** Amniotic band syndrome, Oral rehabilitation, Craniofacial, Unusual.

## Abstract

Amniotic band syndrome (ABS) is a congenital disorder caused by entrapment of fetal parts in fibrous amniotic bands while *in utero*. The syndrome is underdiagnosed and its presentation is variable. The syndrome has been well described in the pediatric, orthopedic and obstetric literature; however, despite the discernable craniomaxillofacial involvement, ABS has not been reported in the dental literature very often. The present report describes a case of a patient with ABS and concomitant dental findings.

**How to cite this article:** Hotwani K, Sharma K. Oral Rehabilitation for Amniotic Band Syndrome: An Unusual Presentation. Int J Clin Pediatr Dent 2015;8(1):55-57.

## INTRODUCTION

Amniotic band syndrome (ABS) is a congenital disorder caused by entrapment of fetal parts in fibrous amniotic bands while *in utero*. The rupture of the amnion has secondary effects on the fetus, which produces malformation and deformation due to interruption of normal morphogenesis. It is also known as ADAM complex.^1^

The syndrome is underdiagnosed and its presentation is so variable that no two cases are exactly identical. Pathogenesis of this defect is probably heterogeneous.^2^ Deformities of the extremities, thorax and craniofacial skeleton and soft tissues occur individually or collectively with varying degrees of severity.^1,2^ The syndrome has been well described in the pediatric, orthopedic, and obstetric literature; however, despite the discernable craniofacial involvement, ABS has not been reported in the dental literature very often.

We present a case of a patient with ABS and concomitant dental findings. A brief review of the syndrome and goals of dental treatment are also discussed.

## CASE REPORT

**Fig. 1:**
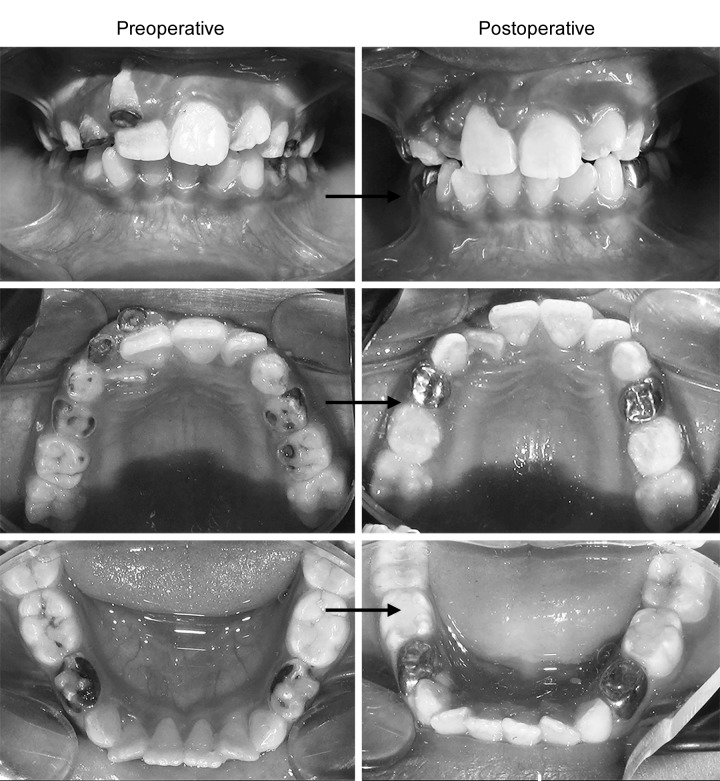
Intraoral photographs

**Fig. 2:**
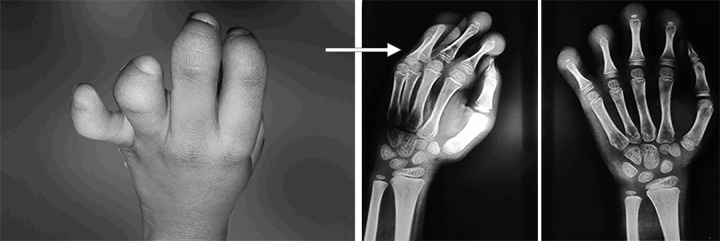
Rudimentary fingers, absent nail bed and weak finger grip (congenital deformity of phalanges of left hand)

An 8-year-old patient reported to the department of pedodontics with the chief complaint of poor esthetics. On clinical examination, it was found that the patient had multiple carious lesions and over-retained deciduous teeth. Patient was a known case of ABS. On further medical evaluation and clinical examination, it was found that the patient had congenital deformity of phalanges of left hand with rudimentary fingers. The nail beds were absent and finger grip was weak (Figs 1 and 2).

On systemic evaluation, patient was found to have frequent gastric regurgitations. A review of relevant medical records showed a history of premature birth with cesarean delivery and traumatic experience of mother during pregnancy.

Intraoral examination showed presence of over-retained 51 and 52. Attrition was noted with 54 and arrested caries with 64. Occlusal caries was present with 65 and 85. Multisurface caries was prominent with 84 and lingual caries with 74. Patient’s dietary history was also recorded and was found to be unbalanced with increased sugar exposures. Intraoral periapical radiographs were taken which confirmed the multiple carious lesions.

A final diagnosis of rampant caries was made. After thorough medical evaluation, consent was obtained from the pediatrician, and patient was evaluated for fitness. A treatment plan was formulated and the rehabilitation resulted in restoration of patient’s oral health considerably (Table 1 and Fig. 1).

## DISCUSSION

The characteristic features described are the constriction of appendages by amniotic bands that may result in:

- Constriction rings around the digits, arms and legs
- Swelling of the extremities distal to the point of constriction
- Amputation of digits, arms and legs^2^

In the present case, congenital deformity of phalanges of left hand and rudimentary fingers were observed. The proposed theories may explain the cause of ABS to some extent. According to amniotic disruption theory proposed by Torpin,^3^ ABS occurs due to a partial rupture of the amniotic sac. The embryonic dysplasia theory proposed in 1930 by Streeter^4^ suggested that abnormal histogenesis causes fetal disruption leading to defective tissue.

**Table 1:** **Table 1:** Phase-wise treatment plan for oral rehabilitation

1. Medical phase		• Evaluation for fitness	
2. Dental rehabilitabtion		• Extraction of root stumps with 51,52	
		• Pit and fssure sealants with 16, 26 and 36, 46	
		• Caries excavation with 54, 64, 74, 84 followed by stainless steel crowns	
		• Glass ionomer restorations with 55, 65, 75 and 85	
3. Preventive measures		• Oral hygiene instructions	
		• Dietary counseling and modification	
		• Periodic recall	

Some studies found connection between ABS and mother’s age, prematurity, abdominal trauma and some drugs.^2,5^ In the present case, a positive history of premature birth and abdominal trauma was established.

The most frequent organs involved in ABS are the fingers and toes, with or without association with cleft lip and palate. Early amniotic rupture, during first 45 days, leads to the most severe craniofacial and visceral malformations. Most often, there are minor defects, such as constriction rings or digit amputations. If bands compress the fetal head or face, different craniofacial disturbances appear: asymmetric face clefts, orbital defects, corneal abnormalities.^1-5^ It is also reported that Infants who are exposed early in pregnancy to miso-prostol may be affected by ABS which is attributed to the process of vascular disruption in limb structures that had formed normally.^6^ However, no such findings could be correlated in the present case.

When we review the treatment aspects, interdisciplinary consulting and work is very often needed. In the present case, proper referral was advocated which ensured a thorough medical evaluation. The dental management for such cases involves a need-based approach as well as patient and parental motivation. Preventive therapies and oral hygiene maintenance form a mainstay of treatment as in many cases, due to upper limb defects, patient is unable to perform adequate brushing of teeth. Thus, there is an urgent need to motivate the patient as well as parents regarding proper preventive home care. In the present case, we presume that avoidance of dental needs by patient and parent, due to already befallen congenital defect, led to the development of rampant dental decay. Also, a history of frequent gastric regurgitation may be a contributory factor toward oral pH environment imbalance. Hence, a planned treatment plan was formulated to include all aspects of dental care, including preventive and restorative therapies in addition to motivation.

The present report, thus, highlights the definite need for reinforcing this preventive and curative oral rehabilitation in ABS patients and also gives acumen into the dental aspects of the syndrome.
